# Accuracy of cause of death data routinely recorded in a population-based cancer registry: impact on cause-specific survival and validation using the Geneva cancer registry

**DOI:** 10.1186/1471-2407-13-609

**Published:** 2013-12-27

**Authors:** Robin Schaffar, Elisabetta Rapiti, Bernard Rachet, Laura Woods

**Affiliations:** 1 Geneva Cancer Registry, Institute for Social and Preventive Medicine, University of Geneva, 55 Boulevard de la Cluse, Geneva, 1205, Switzerland; 2 Cancer Research UK Cancer Survival Group, Department of Non-Communicable Disease Epidemiology, Faculty of Epidemiology and Population Health, London School of Hygiene and Tropical Medicine, London, UK; 3 Centre for Cancer Control and Statistics, Osaka Medical Centre for Cancer and Cardiovascular diseases, Osaka, Japan

**Keywords:** Cause-specific survival, Cause-of-death, Cancer registry, Concordance

## Abstract

**Background:**

Information on the underlying cause of death of cancer patients is of interest because it can be used to estimate net survival. The population-based Geneva Cancer Registry is unique because registrars are able to review the official cause of death. This study aims to describe the difference between the official and revised cause-of-death variables and the impact on cancer survival estimates.

**Methods:**

The recording process for each cause of death variable is summarised. We describe the differences between the two cause-of-death variables for the 5,065 deceased patients out of the 10,534 women diagnosed with breast cancer between 1970 and 2009. The Kappa statistic and logistic regression are applied to evaluate the degree of concordance. The impact of discordance on cause-specific survival is examined using the Kaplan Meier method.

**Results:**

The overall agreement between the two variables was high. However, several subgroups presented a lower concordance, suggesting differences in calendar time and less attention given to older patients and more advanced diseases. Similarly, the impact of discordance on cause-specific survival was small on overall survival but larger for several subgroups.

**Conclusion:**

Estimation of cancer-specific survival could therefore be prone to bias when using the official cause of death. Breast cancer is not the more lethal cancer and our results can certainly not be generalised to more lethal tumours.

## Background

Population-based cancer survival is widely used to evaluate the impact of health care systems in disease management. Net survival is the survival that would be observed if the only possible cause of death were the cancer of interest [1]. Net survival is especially relevant when the cohort of interest become older since the risk of dying from other causes than cancer increases. Net survival is also very useful when comparing subgroups whose mortality due to other causes could be different and therefore lead to biased estimation of the survival contrast.

Two main data designs can be distinguished, the cause-specific and the relative survival designs, according to the availability of information on cause of death. Such information is rarely available in routine, population-based data and net survival is then commonly estimated within the relative survival framework. However, when information about the underlying cause of death is available, net survival can be estimated using the cause-specific approach, in which only deaths from the cause of interest are considered as ‘failures’ , while deaths from other causes are censored. High-quality information on the cause of death is required for each individual patient. This information is commonly available only in clinical trials or hospital series, but the cause-specific approach is sometimes used on population-based data from cancer registries, where the underlying cause of death is derived from death certificates. The underlying cause of death is the “disease or injury which initiated the train of morbid events leading directly to death” or the “circumstances of the accident or violence which produced the fatal injury”. It is codified in The International Classification of Diseases (ICD), which was designed to classify causes of death for statistical tabulation and research. Despite these international rules (developed over 100 years), comparability and accuracy issues still arise. Different medical terminologies, inaccurate completion of the death certificates, misinterpretation or misapplication of the coding rules for selection of the underlying cause of death can cause comparability problems between different geographical areas and/or different periods of time. The validity and accuracy of the reported underlying cause of death may also be incorrect if the clinician’s certification does not accurately reflect the clinical history of events leading to death.

Percy et al. were the first to report that misclassification of the underlying cause of death could bias the mortality trends and therefore the estimation of cancer-specific survival [2]. Many other studies, then, have highlighted the issue of inaccuracy of the cause of death information obtained from death certificates [3-9]. Some studies have shown that the proportion of misclassification can be very high [4,10]. However, one study has suggested that the proportion of misclassification can be lower for screened patients dying from breast cancer [11].

The validity of disease-specific survival is based on the assumption that the underlying cause of death is accurately determined. The Geneva Cancer Registry, which collects all the death certificates of routinely recorded deaths in the Geneva canton (Switzerland), also reviews the cause of death of each registered cancer patient using all the available clinical information relating to the patient’s disease and treatment. This leads to a particular and unique situation in which a second, validated variable defining the cause of death is generated. This second variable is considered to be a more reliable record of the patient’s cause of death and so will be expected to give rise to more accurate estimates of cause-specific survival.

The purposes of this study are (a) to describe the process of recording the cause of death in the Geneva Cancer Registry, (b) to investigate how accurate the routinely recorded cause of death is compared to the validated cause of death derived from clerical review and (c) to examine whether the process of validation leads to differences in the estimates of cause-specific survival.

## Methods

### Data

The data used in this study were obtained from the Geneva Cancer Registry. All women diagnosed with a breast cancer between 1970 and 2009 and resident in Geneva were included in the study.

The Geneva Cancer Registry collects information on incident cancer cases from various sources, including hospitals, laboratories and private clinics, all requested to report new cancer cases. Trained registrars systematically extract information from the medical records and conduct further investigations in the case of missing key data. The variables of interest for this study were cause of death as specified on the death certificate, revised cause of death, age at diagnosis, age at death, year of diagnosis, year of death, social class, stage of the tumour, treatment, sector of care and place of death. The Geneva Cancer Registry has general registry approval by the Swiss Federal Commission of Experts for professional secrecy in medical research (Commission d’experts pour le secret professionnel en matière de recherche medical). This approval permits cancer data collection and its use for research purposes.

### Coding of cause of death

The Geneva Cancer Registry is notified of all deaths occurring in the Geneva canton through three different processes.

First, when a patient dies in the canton of Geneva, a death certificate is compulsorily completed by the clinician certifying the death who reports the primary, secondary and concomitant causes of death. The Geneva Cancer Registry receives photocopies of all these death certificates through the Geneva Health Administration; and links them to the incidence database. The causes of death reported on the death certificates represent the original causes of death.

Meanwhile, once a year, the Federal Office of Statistics (Office Federal de la Statistique, OFS) which is a national publicly-funded organisation collecting death certificates and maintaining a mortality database for the whole of Switzerland provides the Geneva Cancer Registry with a mortality database for the Geneva canton. This is also linked to the incidence database to complete and/or validate the process described above. This leads to the definition of the official cause of death as the underlying cause of death derived from death certificates.

Finally, the Geneva Cancer Registry is provided on an annual basis with information on the vital status of the Canton population by the Cantonal Office of the Population (Office Cantonal de la Population, OCP). OCP is a regional administration that monitors births, deaths, migration, residency and civil partnerships. Only information about the vital status of a patient (deceased or not), or information on whether a person has migrated from Geneva is provided to the Registry. Information on the cause of death is not available within this database.

After all the records are merged, the Cancer Registry registrars then go back to the patient’s charts and review the cause of death according to all the documents available. These include death certificates, autopsy reports, letter at death written by general practitioners and all the patient’s medical notes. By this process the cause of death variable, the *revised cause of death,* is obtained.

Sometimes, the Geneva Cancer Registry is able to obtain information about the occurrence of a death and its cause through the health system (essentially the public health system) before information from death certificates, OFS or OCP. This is particularly so for public sector, where information about the patient’s follow-up is easier to obtain than in the private sector, with which communication is mainly based on mails and willingness of the practitioners.

Some patients leave the canton of Geneva after their diagnosis with cancer, but return and die in Geneva. These individuals are recorded as dead in the OFS database. However, since no additional information on their disease was collected in the Geneva area, they are considered lost to follow up at the point of their departure by the Geneva Cancer Registry.

### Statistical methods

We first examined the agreement between the official underlying cause of death and the reviewed underlying cause of death. We then evaluated the impact of such disagreement on the cause-specific survival estimates.

We used the Kappa statistic to compare concordance between the two cause-of-death variables for all patients who had died (N = 5,065). The Kappa statistic corrects for agreement expected by chance alone. Its values range from 0 to 1; 0 represents no agreement whereas 1 is perfect agreement. We stratified the analysis according to age at diagnosis, age at death, period of diagnosis, period of death, social class, stage, treatment received, sector of care and place of death. Age at diagnosis and age at death were coded into 5 categories (0–49, 50–59, 60–69, 70–79 and 80 and over), whilst four periods were used for the temporal analysis of diagnosis and death (1970–79, 1980–89, 1990–99, 2000–09). Social class was based on the patient’s last job or, if missing, on the patient’s partner’s job. It was divided in four categories (high, medium, low and unknown) [12]. Stage followed the TNM classification [13] with 5 subgroups (stage I, stage II, stage III, stage IV, unknown). We distinguished 5 categories for the treatment each patient received: surgery only, surgery plus adjuvant therapy, hormonal treatment, others (including a mix of different palliative therapies), and an absence of treatment. Only treatments received during the first six months after diagnosis are recorded by the registry according to the IARC rules [14]. Sector of care was defined as private or public sector. We also defined 5 categories of place of death: public hospital, retirement home, private hospital, patient’s home and unknown.

We used variance-weighted least-squares regression to evaluate trends in the Kappa values for sub-groups [15].

We used logistic regression to evaluate the odds of disagreement between the official and revised cause of death, associated with each of the factors listed above.

We also examined the concordance between the official and the revised cause of death as a function of time since diagnosis: patients who died within five years after diagnosis, patients who died after 5 years but before 10 years of follow-up and patients who died after 10 but before 15 years of follow-up. Because of small numbers, patients dying more than 15 years after their diagnosis were not considered.

To estimate the impact of discordance upon cause-specific survival, we derived Kaplan Meier cause-specific survival curves for the whole cohort (N = 10,534) using both official and revised cause of death. In cause-specific survival analyses, patients are classified as presenting the event if they are recorded as dying from their cancer while those who die from other causes are censored at the date of their death. We performed subgroup survival analysis by age group, period of diagnosis, stage of the disease and treatment.

## Results

The cohort consisted of 10,534 women (mean age 61.5 years) diagnosed between 1970 and 2009. Nearly half belonged to the middle social class groups (Table 1). About three quarters of the women were diagnosed at early stage of disease (stage I and II). Almost 90% underwent surgery, associated with adjuvant treatments such as radiotherapy (63%), hormones (44%) and chemotherapy (33%; data not shown).

**Table 1:** Baseline characteristics of the cohort of female breast cancer patients diagnosed in Geneva between 1970 and 2009

		**Overall**		**Deceased**	
		**N**	**%**	**N**	**%**
**Age at diagnosis (mean, SD)**		61,5 (0,14)		66,8 (0,21)	
**Age groups**					
0-50		2′422	23.0	749	14.8
50-59		2′469	23.4	831	16.4
60-69		2′383	22.6	1′055	20.8
70-79		1′949	18.5	1′317	26.0
80+		1′311	12.5	1′113	22.0
**Period of diagnosis**					
1970-79		1′890	17.9	1′576	31.1
1980-89		2′192	20.8	1′552	30.6
1990-99		2′896	27.5	1′302	25.7
2000-09		3′556	33.8	635	12.5
**Socioeconomic status**					
High		1′620	15.4	597	11.8
Middle		5′092	48.3	2′171	42.9
Low		2′390	22.7	1′484	29.3
Unknown		1′432	13.6	813	16.1
**Stage**					
I		3′434	32.6	1′014	20.0
II		4′355	41.3	2′044	40.4
III		1′219	11.6	807	15.9
IV		585	5.6	490	9.7
Unknown		941	8.9	710	14.0
**Treatment**					
Surgery only		1′890	17.9	1′252	24.7
Surgery + adjuvant		7′340	69.7	2′693	53.2
No treatment		473	4.5	421	8.3
Hormones only		547	5.2	448	8.8
Others		284	2.7	251	5.0
**Sector of care**					
Private		5′122	48.6	2′028	40.0
Public		5′412	51.4	3′037	60.0
**Total**		10′534	100.0	5′065	100.0

Among the 5,065 women who have died, the official and the revised underlying cause of death were identical for 4,620 patients (91%) (Table 2). 254 cases (5%) were recorded as dying of breast cancer according to their death certificate but as dying from other causes in the revised data. Among these women, the cause of death was mostly recoded to heart diseases (48%) and other malignant tumours (20%). Conversely, 191 cases (3.8%) were recorded as dying from other causes according to their death certificate but as dying from breast cancer in the revised data. Among these women, the main causes of death reported on their original death certificates were other malignant tumours (40%) or an imprecise code (19%) (Table 2). The overall value of the kappa test was 0.82 (p-value < 0.001).

**Table 2:** Cause of death among women diagnosed with breast cancer in Geneva between 1970 and 2009: effect of reclassification of the official underlying cause of death by the Geneva Cancer Registry

**Cause of death**			**5′065**		
**Concordant**			**4′620**		
*Breast cancer*			*2*′*508*		
*Other cause*			*2*′*112*		
**Discordant**			**445**		
**Distribution of discordant cases**					
**Revised cause of death**	Breast cancer as the official cause of death		**Official cause of death**	Breast cancer as the revised cause of death	
	N	%		N	%
Other tumour	50	19.7	Other tumour	77	40.3
Heart disease	121	47.6	Heart disease	35	18.3
Imprecise code	11	4.3	Imprecise code	37	19.4
Other	72	28.4	Other	42	22.0
	254	100.0		191	100.0

Unadjusted concordance varied greatly between subgroups (Table 3). The concordance was significantly lower with increasing age, from 0.87 for ages 0–49 to 0.74 for ages 80+ (p-value for trend test = 0.008). Similar age-related trends, though not significant, were found among the three subpopulations defined by time since diagnosis. These age-related patterns were much less marked for age at death. Concordance was comparable in all four periods of diagnosis although it tended to be lower in the earlier periods. Concordance was greater for early stage of disease (stage I and II) compared to advanced stage (III and IV), from 0.84 for stage I to 0.63 for stage IV (p-value for trend <0.001). However, the concordance between the two underlying causes of death for women with missing stage (about 14%) tended to be higher than those for stage IV (and stage III). If these records corresponded to advanced diseases, as it is often the case, this stage-related pattern could be greatly attenuated. This pattern was more marked for patients deceased within the first five years after diagnosis. A clear pattern was found according to the type of treatment with higher concordance for complete, with curative intent, treatment (0.83), intermediate concordance for palliative treatment (0.73) and lower concordance for non-treated patients (0.63). This pattern was mostly found among patients who died within five years since diagnosis. We found no association between social class and concordance, but a higher concordance for patients who were monitored (0.86) or who have died (0.85) in the private sector than for those in the public sector (0.80 and 0.76, respectively).

**Table 3:** Concordance by subgroups between the official underlying cause of death and the revised underlying cause of death for women diagnosed with breast cancer in Geneva between 1970 and 2009

	**All data**				**Between 0 and 4 years of follow-up**				**Between 5 and 9 years of follow-up**				**Between 10 and 14 years of follow-up**			
	**N**	**%**	**Kappa**	**SD**	**N**	**%**	**Kappa**	**SD**	**N**	**%**	**Kappa**	**SD**	**N**	**%**	**Kappa**	**SD**
**Overall**	5,065	100.0	0.82	0.01	2,497	100.0	0.76	0.02	1,275	100.0	0.86	0.03	626	100.0	0.83	0.04
**Age at diagnosis**																
0-49	749	14.8	0.87	0.04	317	12.7	0.76	0.06	210	16.5	0.91	0.07	94	15.0	0.92	0.10
50-59	831	16.4	0.87	0.03	380	15.2	0.79	0.05	210	16.5	0.81	0.07	87	13.9	0.90	0.11
60-69	1,055	20.8	0.82	0.03	442	17.7	0.75	0.05	239	18.7	0.87	0.06	146	23.3	0.76	0.08
70-79	1,317	26.0	0.81	0.03	600	24.0	0.73	0.04	339	26.6	0.82	0.05	237	37.9	0.79	0.06
80+	1,113	22.0	0.74	0.03	758	30.1	0.71	0.04	277	21.7	0.79	0.06	62	9.9	0.63	0.12
Trend test				*p = 0.008*				*p = 0.394*				*p = 0.222*				*p = 0.105*
**Age at death**																
0-49	353	7.0	0.80	0.05	248	9.9	0.76	0.06	94	7.4	0.92	0.10	8	1.3	N/A	
50-59	593	11.7	0.86	0.04	350	14.0	0.80	0.05	160	12.6	0.88	0.08	64	10.2	0.96	0.12
60-69	813	16.1	0.79	0.04	427	17.1	0.73	0.05	222	17.4	0.83	0.07	87	13.9	0.86	0.11
70-79	1,105	21.8	0.81	0.03	580	23.2	0.73	0.04	270	21.2	0.85	0.06	130	20.8	0.86	0.09
80+	2,201	43.5	0.77	0.02	892	35.7	0.72	0.03	529	41.5	0.81	0.04	337	53.8	0.73	0.05
Trend test				*p = 0.339*				*p = 0.405*				*p = 0.256*			N/A	
**Period of diagnosis**																
1970-79	1,576	31.1	0.80	0.03	695	27.8	0.72	0.04	354	27.8	0.81	0.05	198	31.6	0.75	0.07
1980-89	1,552	30.6	0.80	0.03	686	27.5	0.69	0.04	387	30.5	0.84	0.05	206	32.9	0.82	0.07
1990-99	1,302	25.7	0.86	0.03	654	26.2	0.81	0.04	366	28.7	0.88	0.05	217	34.7	0.91	0.07
2000-09	635	12.5	0.84	0.04	462	18.5	0.81	0.05	168	13.2	0.90	0.07	5	0.8	N/A	
Trend test				*p = 0.143*				*p = 0.036*				*p = 0.229*			N/A	
**Social Class**																
High	597	11.8	0.81	0.04	281	11.3	0.76	0.06	144	11.3	0.86	0.08	77	12.3	0.77	0.11
Medium	2,171	42.9	0.85	0.02	1,034	41.4	0.78	0.03	553	43.3	0.89	0.04	290	46.3	0.86	0.06
Low	1,484	29.3	0.80	0.03	735	29.4	0.71	0.04	363	28.5	0.82	0.05	177	28.3	0.86	0.07
Unknown	813	16.1	0.79	0.04	447	17.9	0.76	0.05	215	16.9	0.85	0.07	82	13.1	0.73	0.11
Trend testˠ				*p = 0.569*				*p = 0.297*				*p = 0.492*				*p = 0.556*
**Stage**																
Stage I	1,014	20.0	0.84	0.03	280	11.2	0.78	0.06	305	23.9	0.85	0.06	198	31.6	0.84	0.07
Stage II	2,044	40.4	0.83	0.02	901	36.1	0.77	0.03	564	44.2	0.87	0.04	286	46.7	0.84	0.06
Stage III	807	15.9	0.77	0.04	538	21.6	0.74	0.04	175	13.7	0.80	0.08	58	9.3	0.76	0.13
Stage IV	490	9.7	0.63	0.04	427	17.1	0.55	0.05	50	3.9	0.95	0.14	8	1.3	0.38	0.28
Unknown	710	14.0	0.79	0.04	351	14.1	0.70	0.05	181	14.2	0.82	0.07	76	12.1	0.84	0.11
Trend testˠ				*p = 0.000*				*p = 0.000*				*p = 0.963*				*p = 0.272*
**Treatment**																
Surgery only	1,252	24.7	0.83	0.03	436	17.5	0.77	0.05	323	25.3	0.85	0.06	216	34.5	0.82	0.07
Surg + adj.	2,693	53.2	0.86	0.02	1,186	47.5	0.82	0.03	766	60.1	0.87	0.04	367	58.6	0.85	0.05
No treatment	421	8.3	0.63	0.05	317	12.7	0.61	0.06	69	5.4	0.79	0.12	23	3.7	0.47	0.21
Hormones	448	8.9	0.73	0.05	345	13.8	0.70	0.05	88	6.9	0.81	0.11	14	2.2	0.86	0.26
Others	251	5.0	0.65	0.06	213	8.5	0.60	0.07	29	2.3	0.87	0.18	6	1.0	0.57	0.37
**Sector of care**																
Private	2,028	40.0	0.86	0.02	870	34.8	0.81	0.03	550	43.1	0.86	0.04	286	45.7	0.87	0.06
Public	3,037	60.0	0.80	0.02	1,627	65.2	0.73	0.02	725	56.9	0.86	0.04	340	54.3	0.79	0.05
**Period of death**																
1970-79	630	12.4	0.70	0.04	548	21.9	0.71	0.04	82	6.4	0.67	0.11	-	-	N/A	
1980-89	1,196	23.6	0.74	0.03	658	26.4	0.68	0.04	362	28.4	0.84	0.05	149	23.8		
1990-99	1,483	29.3	0.83	0.03	694	27.8	0.78	0.04	377	29.6	0.87	0.05	211	33.7		
2000-09	1,756	34.7	0.88	0.02	597	23.9	0.84	0.05	454	35.6	0.88	0.05	266	42.5		
Trend test				*p = 0.000*				*p = 0.007*				*p = 0.140*			N/A	
**Place of death**																
Public hospital	2,845	56.2	0.76	0.02	1,573	63.0	0.69	0.03	677	53.1	0.82	0.04	315	50.3	0.78	0.06
Retirement home	1,291	25.5	0.83	0.03	570	22.8	0.77	0.04	328	25.7	0.88	0.06	172	27.5	0.79	0.08
Private hospital	150	3.0	0.85	0.08	55	2.2	0.74	0.13	47	3.7	0.77	0.14	24	3.8	1.00	0.20
Home	374	7.4	0.91	0.05	143	5.7	0.94	0.08	113	8.9	0.87	0.09	54	8.3	0.90	0.14
Others	263	5.2	0.96	0.06	122	4.9	0.96	0.09	70	5.5	0.95	0.12	38	6.1	0.92	0.16
Missing	142	2.8	0.92	0.08	34	1.4	0.94	0.17	40	3.1	0.83	0.16	25	4.0	1.00	0.20

ˠTrend test performed without the missing data.

Unadjusted odds ratios of disagreement between the official and the revised underlying causes of death are presented in Table 4 for the overall cohort and for the three subcohorts defined by length of follow-up. The odds of disagreement increased significantly with age at diagnosis (as continuous variable) for all the patients (OR 1.03, 95% CI [1.02; 1.03]) and for the three subcohorts. We observed the same trend when using age at death as continuous variable (OR 1.02, 95% CI [1.01; 1.02] for all patients). Period of diagnosis was not significantly associated with disagreement but we did observe a significant decreasing trend for period of death as a continuous variable for all patients and the subcohorts (OR: 0.97, 95% CI: [0.96-0.98] for all patients). We did not find a significant trend for stage of the disease when considering all patients or the subcohorts defined by follow-up. Patients treated palliatively had significantly higher odds of disagreement (OR: 2.50, 95% CI [1.82; 3.43] for non-treated, 1.76 95% CI [1.26; 2.46] for patients treated with hormones and 1.34, 95% CI [0.86; 2.1] for other palliative treatment). The same trend was observed for the three subcohorts although not statistically significant. Patients treated in the public sector also had a higher risk of disagreement (OR: 1.47, 95% CI [1.20; 1.81] for all patients) as well as those who died in a public hospital. We did not observe differences by social class. We were unable to perform a logistic regression for the subcohort defined by a follow-up time between 10 and 15 years because of the small number of observations (<10) for several variables.

**Table 4:** Univariable logistic regression describing the disagreement by subgroups between the official underlying cause of death and the revised underlying cause of death for women diagnosed with breast cancer in Geneva between 1970 and 2009

				**All data**				**Between 0 and 4 years of follow-up**				**Between 5 and 9 years of follow-up**				**Between 10 and 14 years of follow-up**	
		N	%	OR	95% CI	N	%	OR	95% CI	N	%	OR	95% CI	N	%	OR	95% CI
**Age (continuous)**		5,065	100.0	1.03	[1.02;1.03]	2,497	100.0	1.02	[1.02;1.03]	1,275	100.0	1.02	[1.01;1.04]	626	100.0	1.03	[1.00;1.06]
**Age at diagnosis**																	
60-69		1,055	20.8	1		442	17.7	1		239	18.7	1		146	23.3	1	
0-49		749	14.8	0.54	[0.37;0.81]	317	12.7	0.63	[0.37;1.07]	210	16.5	0.44	[0.17;1.15]	94	15.0	0.25	[0.71;0.88]
50-59		831	16.4	0.64	[0.45;0.92]	380	15.2	0.62	[0.37;1.03]	210	16.5	1.07	[0.50;2.27]	87	13.9	0.37	[0.12;1.12]
70-79		1,317	26.0	1.10	[0.83;1.46]	600	24.0	1.26	[0.85;1.86]	339	26.6	1.40	[0.73;2.67]	237	37.9	0.66	[0.33;1.32]
80+		1,113	22.0	1.54	[1.17;2.04]	758	30.1	1.48	[1.02;2.14]	277	21.7	1.48	[0.76;2.88]	62	9.9	1.12	[0.46;2.76]
**Years (continuous)**				0.99	[0.98;1.00]			0.99	[0.98;1.00]			0.97	[0.95;1.00]			0.94	[0.91;0.97]
**Calendar period**																	
70-79		1,576	31.1	1		695	27.8	1		354	27.8	1		198	31.6	N/A	
80-89		1,552	30.6	1.00	[0.79;1.27]	686	27.5	1.31	[0.95;1.80]	387	30.5	0.84	[0.50;1.43]	206	32.9		
90-99		1,302	25.7	0.69	[0.53;0.91]	654	26.2	0.80	[0.56;1.14]	366	28.7	0.63	[0.36;1.13]	217	34.7		
00-09		635	12.5	0.81	[0.58;1.13]	462	18.5	0.82	[0.56;1.22]	168	13.2	0.52	[0.23;1.16]	5	0.8		
**Social Class**																	
High		597	11.8	1		281	11.3	1		144	11.3	1		77	12.3	1	
Medium		2,171	42.9	0.77	[0.56;1.06]	1,034	41.4	0.87	[0.56;1.33]	553	43.3	0.80	[0.38;1.66]	290	46.3	0.56	[0.24;1.28]
Low		1,484	29.3	1.04	[0.75;1.44]	735	29.4	1.24	[0.80;1.92]	363	28.5	1.30	[0.62;2.71]	177	28.3	0.55	[0.22;1.36]
Unknown		813	16.1	1.11	[0.78;1.59]	447	17.9	1.13	[0.70;1.81]	215	16.9	1.08	[0.47;2.45]	82	13.1	1.05	[0.40;2.74]
**Stage**																	
Stage I		1,014	20.0	1		280	11.2	1		305	23.9	1		198	31.6	1	
Stage II		2,044	40.4	1.24	[0.93;1.66]	901	36.1	1.06	[0.68;1.64]	564	44.2	0.92	[0.53;1.61]	286	46.7	1.24	[0.61;2.52]
Stage III		807	15.9	1.48	[1.06;2.07]	538	21.6	1.03	[0.64;1.64]	175	13.7	1.36	[0.69;2.68]	58	9.3	1.95	[0.74;5.15]
Stage IV		490	9.7	1.25	[0.84;1.85]	427	17.1	0.87	[0.52;1.44]	50	3.9	0.28	[0.04;2.10]	8	1.3	4.74	[0.87;25.87]
Unknown		710	14.0	1.59	[1.13;2.23]	351	14.1	1.51	[0.93;2.44]	181	14.2	1.22	[0.61;2.44]	76	12.1	1.22	[0.45;3.33]
**Treatment**																	
Surgery only		1,252	24.7	1		436	17.5	1		323	25.3	1		216	34.5	1	
Surg + adj.		2,693	53.2	0.78	[0.61;1.01]	1,186	47.5	0.58	[0.40;0.84]	766	60.1	0.85	[0.51;1.41]	367	58.6	0.99	[0.52;1.89]
No treatment		421	8.3	2.50	[1.82;3.43]	317	12.7	1.84	[1.23;2.75]	69	5.4	1.41	[0.58;3.41]	23	3.7	4.41	[1.53;12.75]
Hormones		448	8.9	1.76	[1.26;2.46]	345	13.8	1.34	[0.88;2.03]	88	6.9	1.25	[0.54;2.88]	14	2.2	0.96	[0.12;7.83]
Others		251	5.0	1.34	[0.86;2.10]	213	8.5	1.00	[0.60;1.67]	29	2.3	0.44	[0.06;3.41]	6	1.0	2.50	[0.28;22.71]
**Period of death**																	
continuous				0.97	[0.96;0.98]			0.98	[0.97;1.00]			0.98	[0.96;1.00]			0.94	[0.90;0.97]
70-79		630	12.4	1		548	21.9	1		82	6.4	1				N/A	
80-89		1,196	23.6	1.01	[0.75;1.36]	658	26.4	1.18	[0.84;1.66]	362	28.4	0.60	[0.28;1.30]	149	23.8		
90-99		1,483	29.3	0.69	[0.51;0.93]	694	27.8	0.86	[0.6;1.23]	377	29.6	0.51	[0.24;1.11]	211	33.7		
00-10		1,756	34.7	0.45	[0.33;0.61]	597	23.9	0.65	[0.44;0.96]	454	35.6	0.44	[0.20;0.95]	266	42.5		
**Age at death**																	
continuous				1.02	[1.01;1.02]			1.02	[1.01;1.03]			1.02	[1.01;1.04]			1.03	[1.00;1.05]
60-69		813	16.1	1		427	17.1	1		222	17.4	1		87	13.9	1	
0-49		353	7.0	0.65	[0.39;1.08]	248	9.9	0.66	[0.37;1.18]	94	7.4	0.30	[0.67;1.34]	8	1.3	2.34	[0.24;22.94]
50-59		593	11.7	0.49	[0.31;0.79]	350	14.0	0.51	[0.29;0.90]	160	12.6	0.54	[0.20;1.42]	64	10.2	0.26	[0.30;2.28]
70-79		1,105	21.8	1.13	[0.82;1.56]	580	23.2	1.25	[0.83;1.86]	270	21.2	1.10	[0.55;2.21]	130	20.8	1.22	[0.39;3.77]
80+		2,201	43.5	1.23	[0.93;1.63]	892	35.7	1.46	[1.01;2.10]	529	41.5	1.31	[0.72;2.41]	337	53.8	1.90	[0.72;5.01]
**Place of death**																	
Public hospital		2,845	56.2	1		1,573	63.0	1		677	53.1	1		315	50.3	N/A	
Retirement home		1,291	25.5	0.74	[0.58;0.93]	570	22.8	0.87	[0.64;1.17]	328	25.7	0.71	[0.41;1.22]	172	27.5		
Private hospital		150	3.0	0.59	[0.31;1.14]	55	2.2	0.54	[0.19;1.51]	47	3.7	1.37	[0.52;3.62]	24	3.8		
Home		374	7.4	0.37	[0.22;0.62]	143	5.7	0.20	[0.07;0.54]	113	8.9	0.76	[0.34;1.72]	54	8.3		
Others		263	5.2	0.13	[0.05;0.35]	122	4.9	0.11	[0.03;0.47]	70	5.5	0.17	[0.02;1.23]	38	6.1		
Missing		142	2.8	0.30	[0.12;0.75]	34	1.4	0.21	[0.03;1.53]	40	3.1	0.94	[0.28;3.13]	25	4.0		
**Sector of care**																	
Private		2,028	40.0	1		870	34.8	1		550	43.1	1		286	45.7	1	
Public		3,037	60.0	1.47	[1.20;1.81]	1,627	65.2	1.61	[1.21;2.14]	725	56.9	1.02	[0.66;1.58]	340	54.3	1.60	[0.88;2.91]

**Figure 1:**
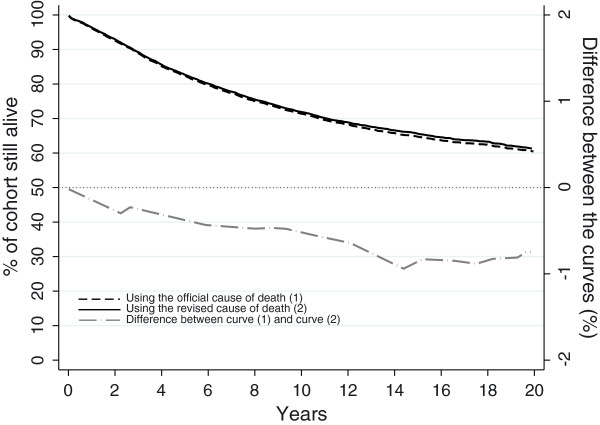
Up-to-20-year cancer-specific survival using 1) the cause of death based on death certificate only and 2) cause of death reviewed by registrars and the absolute difference between them: female breast cancer patients diagnosed between 1970 and 2009.

Figure 1 presents the breast cause-specific survival curves up to 20 years since diagnosis using the two different cause-of-death variables, for all breast cancer patients regardless their final vital status. The survival curves matched almost perfectly, with a difference in 20-year survival lower than 1%. The estimation of proportion of patients alive after twenty years of follow-up when using the official cause of death was 60.51%, 95% CI [59.11; 61.89] and 61.26, 95% CI [59.85; 62.64] when using the revised cause of death.

**Figure 2:**
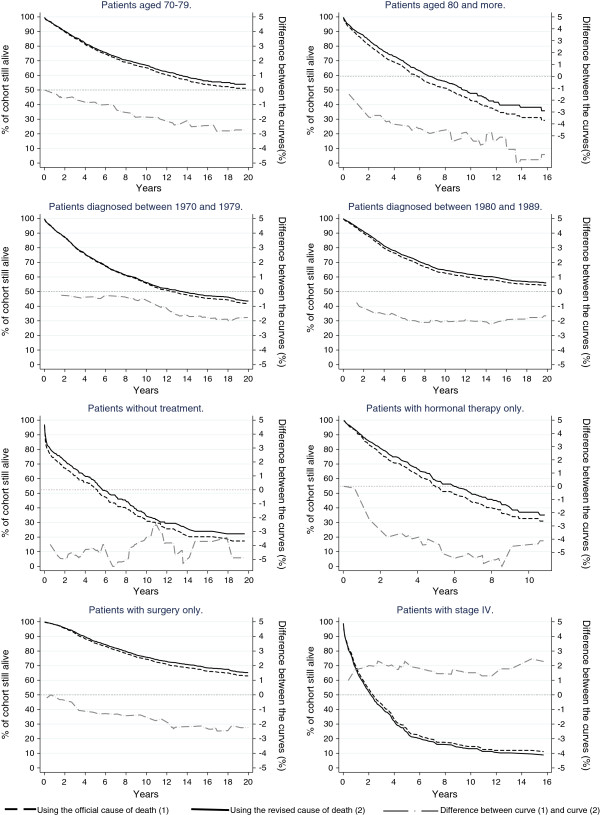
Up-to-20-year cancer-specific survival using 1) the cause of death based on death certificate only and 2) cause of death reviewed by registrars and the absolute difference between them: female breast cancer patients diagnosed between 1970 and 2009. Selected results by co-variables.

We compared cause-specific survival curves estimated with the revised and official underlying cause of death for selected subgroups (Figure 2). We estimated and presented results only if 10 women were remaining in the exposed group and/or the difference between the two curves was larger than 1%. Among patients aged 70–79 the survival at 20-year was 53.9% (95% CI [50.0; 57.6]) when using the revised cause of death and 51.2% (95% CI [47.3; 54.9]) with the official cause of death. The 20-year survival was greater when using the revised cause of death among the two first period of diagnosis. 1.7% and 1.6% difference for 1970–79 and 1980–89 respectively. We also observed a difference for patients treated with surgery. The 20-year survival was 65.2%, 95% CI [62.4; 68.0], based on the revised cause of death and 63.0%, 95% CI [60.1; 65.8] when using only death certificates.

A difference was already present at 10 years for several subgroups. Among patients with no treatment, the estimation was larger for reviewed cause of death with 3.5% difference. Among patients with hormonal therapy, the survival was 4.3% higher, 37.0%, 95% CI [29.5; 44.6] for the reviewed cause of death vs. 32.7%, 95% CI [25.9; 39.6] when using the variable based only on death certificates. In the same way, the survival at 10-year was 5% higher for 80+ when using the reviewed cause of death (47.7%, 95% CI [43.1; 52.1] vs. 42.7%, 95% CI [38.4; 47.0]). Among patients with metastatic tumours, the difference was in the opposite direction: the estimation of 10-year survival was 1.5% higher when using the cause of death based on death certificates only, 14.7%, 95% CI [11.2; 18.7] vs.13.2%, 95% CI [10.0; 16.9] for the reviewed cause of death.

## Discussion and conclusion

Survival statistics derived from routinely collected population-based cancer registry data are key means of reporting progress against cancer. In the Geneva Cancer Registry, in addition to the official underlying cause of death derived from the death certificate, registrars use all the available information in order to establish, where relevant, a revised underlying cause of death which allows evaluation of the accuracy of death certification.

This study describes both processes of recording the cause of death and shows their impact upon estimated survival rates from breast cancer.

The overall concordance between the official and the revised underlying cause of death was high. Differences were only present for 8.8% of the deceased patients representing 4.2% of the entire cohort. This is consistent with the study conducted by Goldoni et al. [11] in 2009 who reported 4.3% misclassification among their cohort. The official underlying cause of death was revised to breast cancer in 191 women (3.8% of those who have died) according to the cancer registry registrars; the underlying cause of death of these women had mainly been coded to other tumours. This could be explained by the presence of metastases that may have misled the certifying doctor about the location of the primary cancer and leads to differences in cause-specific survival estimation among metastatic patients (Figure 2).

On the other hand, most of the 254 women (5.0% of the patients who have died), coded as breast cancer deaths on the death certificates and considered as deaths from other causes from the registry, have been attributed to heart disease. Most of these women were elderly patients diagnosed during 1970–89. At that time the guidance for death certification among cancer registries was not to emphasize the cancer as a cause of death [16]. This might explain a tendency to recode the cause of death from cancer to heart diseases among elderly.

Our results based on Kappa statistic and on logistic regression showed that disagreement was greater among elderly women, patients with advanced disease and patients receiving palliative treatment. This suggests that less attention is given by doctors certifying death to the underlying cause of death for patients who are more likely to die. Concordance is also lower within the first five years after diagnosis, suggesting that more accurate information is available to the registrars assessing the true underlying cause of death during a shorter period of follow-up.

We also observed increasing concordance in successive calendar periods of death. Since this variable closely represents the year in which the review took place, several explanations may apply. First, the Geneva Cancer Registry may have less information in more recent times. This seems unlikely since more linkages have been set up over time with the health system in the canton, allowing a greater exchange of data. More likely, the accuracy of death certificates has improved over time which has led to more confidence in the official coding supplied on death certificates.

It is legitimate to ask why the reliability of cause of death reported on the death certificates may be questioned at all. It can be argued that the general practitioner responsible for the patient is the person most likely to be aware of the underlying cause of death insofar as they are aware of all the clinical information and also often know the patients personally. However, this advantage is not always capitalised on. Physicians are more likely to misclassify the cause of death than a trained registrar [4,10,17-19]. The general practitioner is not always concerned about the epidemiological information they are providing, and may not be aware of the international rules of WHO about the coding of the cause of death. Moreover, the general practitioner often receives the results of the autopsy after the death certificate has been issued and therefore does not take into account the report when certifying the death. The registrars of the Geneva Cancer Registry, on the other hand, are able to access all pathological and histological information and/or the clinical information for most cases and review the cause of death only in the light of the autopsy reports. In addition, the registrars are more experienced with epidemiological data and its coding.

James [20] showed that coding the cause of death using death certificates only, in isolation from all other available information, led to biased interpretations of the cause of death. Our study tends to legitimate the process of verification that is performed in the Geneva Cancer Registry and induces that the resulting estimation of survival is more accurate.

Both methods aim to assign as best as possible the cause of death and none of them can be considered as the gold standard. We nevertheless consider the revised cause of death as more accurate insofar as additional information is available to experienced registrars but not necessarily to the practitioner completing the death certificate.

Overall, the revised underlying cause of death did not have a major impact on the cause-specific survival up to 20 years. However, important differences appeared in several subgroups suggesting that using the official underlying cause of death could lead to biased estimation of cause-specific survival in some populations.

The main limitation of this study relates to the proportion of women who have died for whom information other than the death certificate was available. The more information available, the more likely it is that we will be able to find discordance. High concordance reflects either lack of additional information available to correct the official cause of death, or that the death certificates define the cause of death fairly well. However, among our cohort of 5,062 deceased patients, a high percentage was monitored and/or passed away in the public sector of care, where access to information about cause of death is more readily available. We therefore assume that information enabling review of the underlying cause of death was available for the great majority of women who had died and that the overall high concordance between official and revised underlying cause of death is real.

Moreover, the number of deaths in the cohort influences the discordance. The more deaths, the more likely it is to find differences between the two causes-of-death and then the concordance. This state is confirmed in our study with a higher discordance among elderly. Breast cancer is not the more lethal cancer and our results can certainly not be generalised to other tumour localisations.

The Geneva Cancer registry data represent a unique opportunity to review the accuracy of the cause of death recorded on a death certificate by comparing it to all the available information in the health system. We observed that the overall concordance with the cause of death found on the death certificates is fairly high. More particularly, the impact on estimates of cause-specific survival is very small overall, although analyses in subgroups show larger differences, suggesting that misclassification of the underlying cause of death could lead to biased estimation of differences or trends in cause-specific survival.

## Competing interest

There are no conflicts of interest to declare.

## Authors’ contributions

All authors contributed to the manuscript. RS conducted the analysis and the writing under the supervision of LW and BR. BR, LW and ER all reviewed the paper and made final corrections. All authors read and approved the final version of the manuscript.

## Pre-publication history

The pre-publication history for this paper can be accessed here:

http://www.biomedcentral.com/1471-2407/13/609/prepub
